# Effects of Cycloergometer on Cardiopulmonary Function in Elderly Patients after Coronary Artery Bypass Grafting: Clinical Trial

**DOI:** 10.1155/2024/3808437

**Published:** 2024-09-20

**Authors:** André Luiz Lisboa Cordeiro, Hayssa De Cássia Mascarenhas Barbosa, Kaliane Pereira Vaz, Layla Souza E. Souza, Laura Brandão De Souza, Thayná De Oliveira Matos, André Raimundo França Guimarães

**Affiliations:** ^1^ Bahiana School of Medicine and Public Health, Salvador, Bahia, Brazil; ^2^ Nobre University Center, Feira de Santana, Bahia, Brazil; ^3^ Nobre Institute of Cardiology, Feira de Santana, Bahia, Brazil

## Abstract

**Introduction:**

Despite all the improvements in surgical and anesthetic techniques, this procedure is still associated with pulmonary and cardiovascular complications in the postoperative period, and early rehabilitation, done through the use of cycloergometer, can minimize such complications, besides reducing the length of hospital stay.

**Objective:**

Therefore, the aim of the study was to assess the impact of cardiovascular exercise on lung function, respiratory muscle strength, and functional capacity in elderly patients after heart bypass surgery.

**Methods:**

To this purpose, a randomized and controlled clinical trial was conducted. Research participants were randomized to the cycle ergometer group (CEG) or to the control group (CG). The CG was managed based on the institution's protocol. The CEG also carried out all the activities of the control group, but there was the inclusion of cycle ergometry through a device built by the researchers. Pulmonary function (vital capacity (VC) and peak expiratory flow (PEF)), ventilatory muscle strength (maximum inspiratory pressure (MIP) and maximal expiratory pressure (MEP)), and functional capacity (six-minute walk test) were evaluated before surgery, at ICU, and hospital discharge.

**Results:**

During the research period, 122 patients were evaluated, 61 in each group. The MIP of the cycle ergometry group was higher at discharge from the ICU 95% CI 8 (5.46 to 10.54) and at hospital discharge 95% CI 14 (16.89 to 11.11). MEP was higher in the cycle ergometry group at discharge from the ICU with 95% CI 6 (8.18 to 3.82) and at hospital discharge with 95% CI 9 (11.69 a 6.31). Vital capacity at ICU discharge with 95% CI 6 (7.98 to 4.02) and at hospital discharge with 95% CI 7 (8.98 to 5.02), as well as peak flow at ICU discharge with 95% CI 43 (75.27 to 10.73), showed relevance, being higher in the group that used the cycle ergometer. The CEG showed improvement in functional capacity at the time of hospital discharge with a 95% CI 56 (30.37 to 81.63).

**Conclusion:**

We conclude that application of cycloergometry after CABG decreases the loss of pulmonary function, muscle strength, and functional capacity. This trial is registered with RBR-39yrht6.

## 1. Introduction

As the population age increases, the occurrence of cardiovascular diseases has increased and one of the treatments used is surgery, such as coronary artery bypass grafting. Despite all the improvements in surgical and anesthetic techniques, this procedure is still associated with pulmonary and cardiovascular complications in the postoperative period, and early rehabilitation, done through the use of cycloergometer, can minimize such complications, besides reducing the length of hospital stay [1, 2].

According to the World Health Organization (WHO), in 2016, the mortality of people with cardiovascular disease averaged 17.9 million, globally it characterized 31% of deaths [3]. Obesity, sedentary lifestyle, and hypertension are risk factors for comorbidities with advancing age. In 2013, the Brazilian Institute of Geography and Statistics (IBGE) counted by age groups with some cardiovascular disease, represented about 11.9% of people aged 69 to 74 years and 13.7% aged 75 years or more [4].

It is known that aging is a natural process that is characterized by a set of changes in the respiratory system due to physiological changes such as the decrease in the elastic recoil of the lungs and anatomical changes in the rib cage leading to a decline in independence and interfering with the elderly's quality of life [5–7].

The alterations generated in the respiratory system caused by cardiac surgery may be related to factors arising from the surgical act such as anesthesia, cardiopulmonary bypass (CPB), surgical incision, patient's hemodynamics, type of surgery, duration of the procedure, pain, and thoracic drainage, being a limiting factor in the worsening of physical conditioning by triggering significant changes in volumes and pulmonary capacity, as well as ventilatory and peripheral muscle strength [8, 9].

Coronary artery bypass grafting (CABG), also known as bypass grafting or coronary bypass, is a commonly performed intervention to restore blood flow to the heart in patients with severe coronary artery disease (CAD). However, despite advances in surgical technique, pulmonary and cardiovascular complications still pose significant concerns after CABG. Studies have shown that the incidence of pulmonary complications, such as atelectasis and pneumonia, ranges from 5% to 30%, while cardiovascular complications, including cardiac arrhythmias and myocardial infarction, occur in up to 20% of patients undergoing CABG [7, 8]. These complications can prolong hospital stays, increase treatment costs, and negatively impact patients' postoperative quality of life. Therefore, understanding and addressing these complications effectively is crucial to optimize outcomes after CABG.

Thus, cardiac rehabilitation aims to help patients recover and restore their preoperative conditions, and the cycloergometer is a therapeutic modality used for early establishment in the postoperative period [7]. The study by Borges et al. reports that patients who performed cycle ergometer exercises during hospitalization from cardiac surgery achieved recovery and maintenance of functional capacity [8].

The cycloergometer is a stationary device that promotes the recovery of functional capacity during the postoperative period of cardiac surgery with relevance in respiratory and motor mechanics, being determinant for the reduction of mechanical ventilation time, increase in muscle strength, improvement in functional capacity, and reducing complications at the bedside, it is an accessible device with high acceptance by patients that does not alter blood pressure during or after exercise [10]. It is a safe and effective method in upper and lower limbs generating hemodynamic changes compatible with exercise [11].

Cycloergometry plays a key role in the rehabilitation of patients after heart surgery, offering a safe and effective approach to improve functional capacity and quality of life. Through controlled exercise on a stationary bike, patients can carry out a personalized physical training program aimed at progressively recovering cardiorespiratory function, strengthening muscles, and increasing endurance. In addition, cycle ergometry makes it possible to closely monitor the cardiovascular response during exercise, adjusting the intensity according to the patient's individual tolerance, thus contributing to the prevention of complications and the promotion of a faster and more complete recovery. All these effects may be associated with a reduction in pulmonary complications and length of hospital stay.

With this study, we sought to test the hypothesis that the use of a cycle ergometer in elderly patients after cardiac surgery can minimize the loss of cardiopulmonary function, muscle strength, and functional capacity.

Despite the evidence, the available studies do not limit the use of the cycle ergometer in the elderly, and this work is directed to this population. Therefore, the aim of the present study is to assess the impact of cardiovascular exercise on lung function, respiratory muscle strength, and functional capacity in elderly patients following cardiac bypass surgery.

## 2. Methods

### 2.1. Study Design

This is a randomized controlled trial conducted with patients undergoing coronary artery bypass grafting at Instituto Nobre de Cardiologia in Feira de Santana—Bahia, from November 2021 to December 2022. This study is registered in the Brazilian Registry of Clinical Trials (ReBEC) under number RBR-39yrht6.

### 2.2. Inclusion and Exclusion Criteria

The following inclusion criteria were used: Individuals of both genders with coronary artery disease (CAD), over 60 years of age and undergoing coronary artery bypass grafting with cardiopulmonary bypass and median sternotomy. The following were excluded: use of intra-aortic balloon, surgical reintervention, death, valve disease, previous pneumopathy, those who did not understand how to perform the proposed techniques, those who presented hemodynamic instability during the evaluation or application of the cycle ergometer, and physical limitations such as amputation that compromised the performance of the exercises.

### 2.3. Sample Calculation

To perform the sample calculation, we conducted a pilot study with 10 patients. We verified a standard deviation of 76 meters, in the six-minute walk test, based on the pilot in the control group and 116 meters with respect to the standard deviation in the cycloergometer group. We use a difference of 50 that relates to the clinically relevant distance [9]. For an alpha of 5% and aiming to achieve 80% power, 122 patients were needed, 61 in each group.

### 2.4. Ethical Aspects

Our study was submitted and approved by the Ethics and Research Committee of Faculdade Nobre de Feira de Santana obtaining opinion number 2.150.427. All participants signed an informed consent form. The consent form was signed preoperatively in a specific room. The form was administered by an independent researcher.

### 2.5. Randomization

The study was blinded by an independent hospital employee who prepared opaque sealed envelopes containing either number 1 (intervention group) or number 2 (control group). The patient then chose one of the opaque envelopes and was assigned to that group by the same employee. An independent person not involved in this study possessed the randomization. The researcher who collected the data had no knowledge of the group allocations.

### 2.6. Study Protocol

The control group was managed based on the institution's protocol, which consists of the application of noninvasive ventilation, breathing exercises, kinesiotherapy, and walking. Care was provided three times a day while in the intensive care unit (ICU), and twice in the open unit. The total time of care was 20 to 30 minutes.

**Figure 1:**
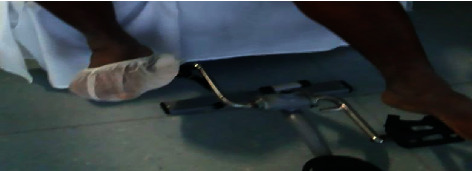
Device built by the researchers.

The CEG also performed all the activities of the control group, but cycloergometry was included through a device built by the researchers (Figure 1). The cycle ergometer was produced by the researchers themselves. There were two suspended rods, upper castings, and two pedals. The participants were instructed to pedal continuously, without adding weight to the equipment, for 10 minutes, all performed on the lower limbs. In both groups, vital signs were continuously assessed. The care was also performed three times a day while in the ICU, and twice in the open unit, totaling 30 to 40 minutes of activity when added to the unit's protocol. Pulmonary function, ventilatory muscle strength, and functional capacity were assessed before surgery, at ICU discharge, and in the hospital. The evaluation of the patients was performed by a trained, blinded examiner.

Clinical and surgical characteristics such as diabetes mellitus, hypertension, dyslipidemia, acute myocardial infarction, and sedentary lifestyle were collected. All these comorbidities were known through the medical records of each patient, with the exception of sedentarism, where the International Physical Activity Questionnaire (IPAQ) was applied in the long format, which evaluates 27 questions related to physical activities performed in a normal week, with light, moderate, and vigorous intensity, lasting 10 minutes continuously, divided into four categories of physical activity such as work, transportation, household activities, and leisure. Those who did not perform any physical activity for at least 10 minutes continuously during the week were considered to be inactive [10].

### 2.7. Variables Evaluated

The preoperative evaluation of the inspiratory muscle strength (maximum inspiratory pressure (MIP)) was performed using an Indumed® analog manometer. During the evaluation, it was requested a maximum expiration up to the residual volume and then a maximum and slow inspiration up to the total lung capacity, and this test was done through the method with the unidirectional valve, allowing a flow through a one-millimeter orifice in order to exclude the action of the buccinator, and repeated three times, using the highest value reached, provided that this value was not the last. The expiratory muscle strength (maximal expiratory pressure (MEP)) was evaluated using the same device, and the patient was instructed to take a maximum inspiration until he reached his total lung capacity (TLC), the mask was placed, and after that a maximum expiration was requested until the residual capacity was reached. The test was repeated 3 times, and the result with the highest value was considered, but it could not be the last one [11].

For vital capacity evaluation, a face mask was used, connected to the expiratory branch of the analog ventilometer (Ferraris–Mark 8 Wright Respirometer, Louisville, CO, USA), and the patient was instructed about all the test phases. The ventilometer was unlocked, zeroed, and then the face mask was placed on the subject's face. He took a deep inspiration until his total lung capacity (TLC) was reached, followed by a slow and gradual exhalation until his residual volume was reached. After that the ventilometer was locked and the result observed and noted. The test was repeated 3 times, considering the result with the highest value [12].

Peak expiratory flow was assessed using the Mini Wright® peak flow. During the evaluation, the patient was seated, with his head in a neutral position and a nose clip to prevent air escaping through the nostrils. The patient made a deep inspiration up to total lung capacity, followed by a forced expiration with the mouth in the device. After three measurements, the highest value was chosen, and there could be no difference greater than 40 liters between measurements [12].

The six-minute walk test (6MWT) assessed functional capacity, evaluation of response to intervention, risk of falling, and predictor of mortality; it is a low cost test, easy to accept and understand. The application of the test was performed by a trained team, the volunteers were instructed on how the 6MWT works, and the test was performed in a flat, free, unobstructed 30-meter long corridor for 6 timed minutes. After performing the 6MWT, the patient sat in a chair and the following variables were monitored again: blood pressure, heart rate (HR), respiratory rate (RR), peripheral oxygen saturation (SpO_2_) through pulse oximeter, those who presented one of the variables outside the parameter did not participate in the test [13].

### 2.8. Statistical Analysis

The Statistical Package for Social Sciences version 20.0 software was used for data analysis. Normality was assessed using the Shapiro–Wilk test. Data were expressed as mean and standard deviation. The Chi-square test was used to compare categorical variables. For comparison of numerical variables between groups, the independent Student's *t*-test was used. For the intragroup analysis between the different moments, the paired Student's *t*-test was used. A 95% confidence interval was also measured. To be considered significant, *p* should be less than 5%.

## 3. Results

**Figure 2:**
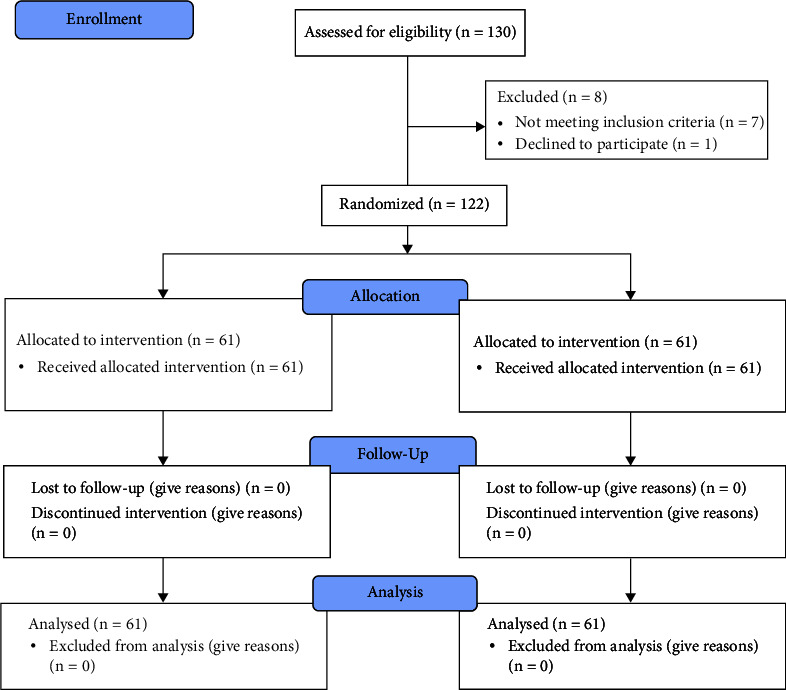
Flowchart on adding the research participants.

During the study period, 122 patients were evaluated (Figure 2), with a mean age of 61 ± 6 years. The most prevalent gender was male with 76 (62.29%), most patients were sedentary with 89 (76.22%), and the most prevalent comorbidity was SAH with 64 (52.45%). We highlight the length of hospital stay, and the cycloergometer group spent less time in the hospital (7 ± 1 days) when compared to the control group (10 ± 3 days). The other clinical and surgical data are presented in Table 1.

**Figure 3:**
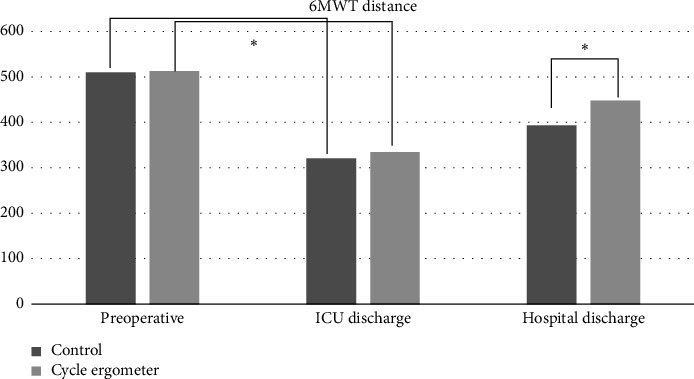
Behavior of functional capacity in the preoperative period, ICU discharge, and hospital discharge among the groups studied. 6MWT: 6-minute walk test; ICU: intensive care unit.

The behavior of the functional capacity in the groups studied is shown in Table 2. The group that performed cycloergometry showed an improvement in functional capacity compared to the control group at hospital discharge with 95% CI 56 (30.37 to 81.63), while in the preoperative period and at ICU discharge, there was no statistical difference between the groups.  Figure 3 shows the behavior of functional capacity at the three moments of the study, comparing the two groups.

In Table 3, we have the comparison in relation to lung function and muscle strength, and we verified that the MIP of the cycloergometry group was higher than that of the control group at ICU discharge 95% CI 8 (5.46 to 10.54) and at hospital discharge 95% CI 14 (16.89 to 11.11). The MEP was higher in the cycloergometry group at ICU discharge with 95% CI 6 (8.18 to 3.82) and at hospital discharge with 95% CI 9 (11.69 to 6.31). The vital capacity at ICU discharge with 95% CI 6 (7.98 to 4.02) and at hospital discharge with 95% CI 7 (8.98 to 5.02), as well as the peak flow at ICU discharge with 95% CI 43 (75.27 to 10.73) showed relevance, being higher in the group that used the cycloergometer. The other values and comparisons were not statistically significant.

## 4. Discussion

Postoperatively, there is a decrease in functional capacity due to muscle weakness, pain, and the patient's fear of performing activities during the hospital stay [14]. The application of early rehabilitation can be used as a protective strategy for these patients [15]. Among the various forms of mobilization, we find cycloergometry. In our study, we verified that this tool was able to reduce the loss in the patients studied. This result can be attributed to the improvement in cardiorespiratory conditioning, cardiac function, and muscle strength, whether respiratory or peripheral.

Hirschhorn et al. [16] reported in their study that the use of moderate intensity of cycloergometry was effective for restoring functional capacity in the postoperative period of coronary artery bypass graft surgery, being well tolerated, emphasizing that the cycloergometer is a viable option in clinical practice due to its ability to measure and record hemodynamic parameters, of pulse oximetry, measurement, and prescription of the imposed exercise load and total dose of exercise, having the advantage of being able to be interrupted immediately if the patient presents hemodynamic or respiratory impairment, and being a good option for patients with impaired gait. In the present study, we identified similarities with the cycloergometry group, which showed an improvement in functional capacity at hospital discharge.

Our group has already demonstrated that the application of respiratory muscle training reduces the loss of functional capacity in patients after cardiac surgery; this resource is also viable, since its application can be performed in any environment, but the difference is that cycloergometry ends up having a much more systemic repercussion, increasing not only respiratory muscle strength but can also have an impact on the increase in peripheral muscle strength.

The difference with the cycle ergometer is its viability, since it can be applied in more restricted environments and patients. Walking is a tool that aims at improvement, but it needs space to occur, unlike cycloergometry. Just like virtual reality, which despite its benefits requires a large budget for technology and is not feasible in all public hospitals, cycloergometry is a more accessible and less expensive tool.

In the walking test, the difference between the groups at hospital discharge was 56 meters, which represents a significantly important minimal difference, since for this population, an increase of 25 meters is considered clinically important, generating impacts on the patient's life [17].

Patients restricted to bed during the postoperative period evolve with contractures, atrophy, decreased mobility, and muscle strength [14]. These complications make it impossible to use some conducts; being primordial, the early intervention with the cycloergometer because they favor a better cardiorespiratory functionality, reducing the loss of muscle strength and gain of range of motion [18]^.^

According to Dantas et al. [13], the cycloergometer can be used in the initial phase of physical and functional rehabilitation since the patient had cardiorespiratory stability in order to restore peripheral muscle strength, gain range of motion, cardiorespiratory conditioning, and contribute to increasing mobility at the time of discharge. Another relevant factor mentioned is the importance of safety when performing cycloergometry, being necessary to evaluate intrinsic and extrinsic factors to the patient and the participation of the multidisciplinary team in the process. Corroborating these findings, Burtin et al. report that exercises performed early can improve the recovery of functional capacity and strength of peripheral muscles at hospital discharge.

Gardenghi et al. [11] verified that the application of cycloergometer for the upper limbs does not generate significant hemodynamic impact, not being necessary to increase the vasoactive drugs; this result can be beneficial since it makes the application of cycloergometry safe, being feasible in an early manner, influencing in the reduction of hospital stay and in the loss of muscle strength.

Almeida et al. [19] verified in their study a significant increase in the expiratory peak flow being generated by the increase in ventilation that occurs due to the body's need to generate energy during exercise for muscle contraction during the exercises performed sitting and standing, generating an increase in the pressure gradient. In our study, the cycloergometer group had a significant increase in peak expiratory flow (PEF) at ICU discharge, possibly due to increased oxygen consumption during exercise by increasing lung ventilation. At hospital discharge, PEF values between the groups were the same, probably due to a decrease in the number of times, the patient cycled in the open unit compared to the ICU.

The effect of anesthesia limits the expansion of the rib cage together with pain, leading the patient to adopt shallow breathing, reducing lung compliance, and increasing the risk of atelectasis [20]. In our study, the cycloergometer proved to be effective at hospital discharge, since it generated an increase in inspiratory pressure and vital capacity after use, and may reduce respiratory complications in the postoperative period.

Several studies presented here have reported on the cardiovascular changes resulting from the use of the cycle ergometer, such as the increase in the heart rate, respiratory rate, and blood pressure early in the exercise. These changes also imply lung function and, consequently, alter vital capacity. This becomes a positive fact as it is a response to the increased oxygen in the body's systems and tissues [21].

The present study has limitations that must be considered. The study did not investigate the relationship of cardiorespiratory changes present during exercise performance and the use of vasoactive drugs with the findings. Another limitation was not evaluating the safety of cycloergometer use and its effects after hospital discharge. Another point is not having evaluated the peripheral muscles, their outcomes, and lack of blinding.

These results suggest that intervention with cycle ergometry contributes to a faster and more efficient recovery, minimizing typical postsurgery functional losses. The clinical implications are substantial, as the incorporation of the cycle ergometer into the post-CABG rehabilitation protocol can improve the overall recovery of patients, reducing respiratory complications and promoting a more agile recovery, which can, in turn, reduce the length of hospital stay and improve patients' quality of life.

## 5. Conclusion

Based on the observed data of the results, we verified that the application of cycloergometry after cardiac surgery has a positive impact in reducing the loss of functional capacity, evaluated through the six-minute walk test. In addition, there was a decrease in the loss of lung function and inspiratory and expiratory muscle strength, assessed through vital capacity, MIP, and MEP, respectively.

## Figures and Tables

**Table 1:** Clinical and surgical data of the patients studied.

Variable	Control group (*n* = 61)	Cycle ergometer group (*n* = 61)	*p*
Age (years)	59 ± 5	62 ± 6	0.54^a^
Gender			0.87^b^
Male	39 (64%)	37 (61%)	
Female	22 (36%)	24 (39%)	
BMI (kg/m^2^)	25 ± 3	26 ± 2	0.68^a^
Smoking history	6 (10%)	7 (11%)	0.71^b^
Ejection fraction (%)	56 ± 7	54 ± 5	0.68^a^
Level of physical activity			0.76^b^
Active	15 (25%)	18 (30%)	
Sedentary	46 (75%)	43 (70%)	
Comorbidities			
SAH	33 (54%)	31 (51%)	0.68^b^
DM	21 (34%)	23 (38%)	0.78^b^
DLP	15 (25%)	15 (25%)	0.93^b^
Surgery time (hours)	4.1 ± 1.2	4.4 ± 1.6	0.66^a^
CPB time (min)	88 ± 12	92 ± 10	0.42^a^
Length of stay in ICU (days)	2 ± 1	2 ± 2	0.64^a^
Length of hospital stay (days)	10 ± 3	7 ± 1	0.04^a^
MV time (hours)	7 ± 2	8 ± 2	0.76^a^
Number of drains	2 ± 1	2 ± 1	0.92^a^
Number of grafts	2 ± 1	2 ± 1	0.94^a^
Total number of physical therapy sessions	1.342	976	0.03^a^

^a^Independent Student's *t*-test; ^b^Chi-square; BMI: body mass index; ECC: extracorporeal circulation; SAH: systemic arterial hypertension; DM: diabetes mellitus; DLP: dyslipidemia; ICU: intensive care unit; MV: mechanical ventilation.

**Table 2:** Data related to functional capacity among the groups studied.

Variable	Cycle ergometer group (*n* = 61)	Control group (*n* = 61)	IC 95%	*p* ^a^
6MWT (meters)				
Preoperative	512 ± 70	510 ± 67	2 (−26.56^a^ 22.56)	0.82
ICU discharge	333 ± 61	321 ± 77	12 (−36.90^a^ 12.90)	0.32
Hospital discharge	448 ± 72	392 ± 71	56 (30.37^a^ 81.63)	<0.01

^a^Independent Student's *t*-test. 6MWT: six-minute walk test.

**Table 3:** Data related to pulmonary function and muscle strength among the groups studied.

Variable	Cycle ergometer group (*n* = 61)	Control group (*n* = 61)	IC 95%	*p* ^a^
MIP (cmH_2_O)				
Preoperative	114 ± 8	115 ± 7	−1 (3.69^a^ −1.69)	0.83
ICU discharge	75 ± 6^b^	67 ± 8^b^	8 (5.46^a^ 10.54)	<0.01
Hospital discharge	91 ± 7	77 ± 9^c^	14 (16.89^a^ 11.11)	<0.01
MEP (cmH_2_O)				
Preoperative	95 ± 9	94 ± 8	1 (−4.05^a^ 2.05)	0.72
ICU discharge	62 ± 7^b^	56 ± 5^b^	6 (8.18^a^ 3.82)	0.04
Hospital discharge	77 ± 8	68 ± 7^c^	9 (11.69^a^ 6.31)	<0.01
VC (ml/kg)				
Preoperative	52 ± 7	54 ± 6	−2 (−0.34^a^ 4.34)	0.76
ICU discharge	38 ± 6^b^	32 ± 5^b^	6 (7.98^a^ 4.02)	0.03
Hospital discharge	47 ± 5	40 ± 6^c^	7 (8.98^a^ 5.02)	0.04
PEF (L/min)				
Preoperative	430 ± 111	420 ± 102	10 (−48.22^a^ 28.22)	0.69
ICU discharge	354 ± 91^b^	311 ± 89^b^	43 (75.27^a^ 10.73)	0.02
Hospital discharge	388 ± 99	370 ± 91	18 (52.09^a^ 16.09)	0.34

^a^Student's *t*-test independent with *p* < 0.005 comparing groups. ^b^Student's *t*-test paired with *p* < 0.005 comparing preoperative with ICU discharge. ^c^Student's *t*-test paired with *p* < 0.05 comparing preoperative with hospital discharge; CI: confidence interval; IMT: inspiratory muscle training; MIP: maximal inspiratory pressure; MEP: maximal expiratory pressure; VC: vital capacity; PEF: peak expiratory flow.

## Data Availability

The authors agree on the availability of data.
